# Supplementary material for: Representation of internal speech by single neurons in human supramarginal gyrus
Source: Nat Hum Behav. 2024 May 13;8(6):1136–49. doi: 10.1038/s41562-024-01867-y (PMC11199147; doi:10.1038/s41562-024-01867-y)
Supplement: Supplementary file 1 — Supplementary Figs. 1–5. [file 41562_2024_1867_MOESM1_ESM.pdf]

nature human behaviour

Article

https://doi.org/10.1038/s41562-024-01867-y

Representation of internal speech by single neurons in human supramarginal gyrus

In the format provided by the authors and unedited

Supplementary Information

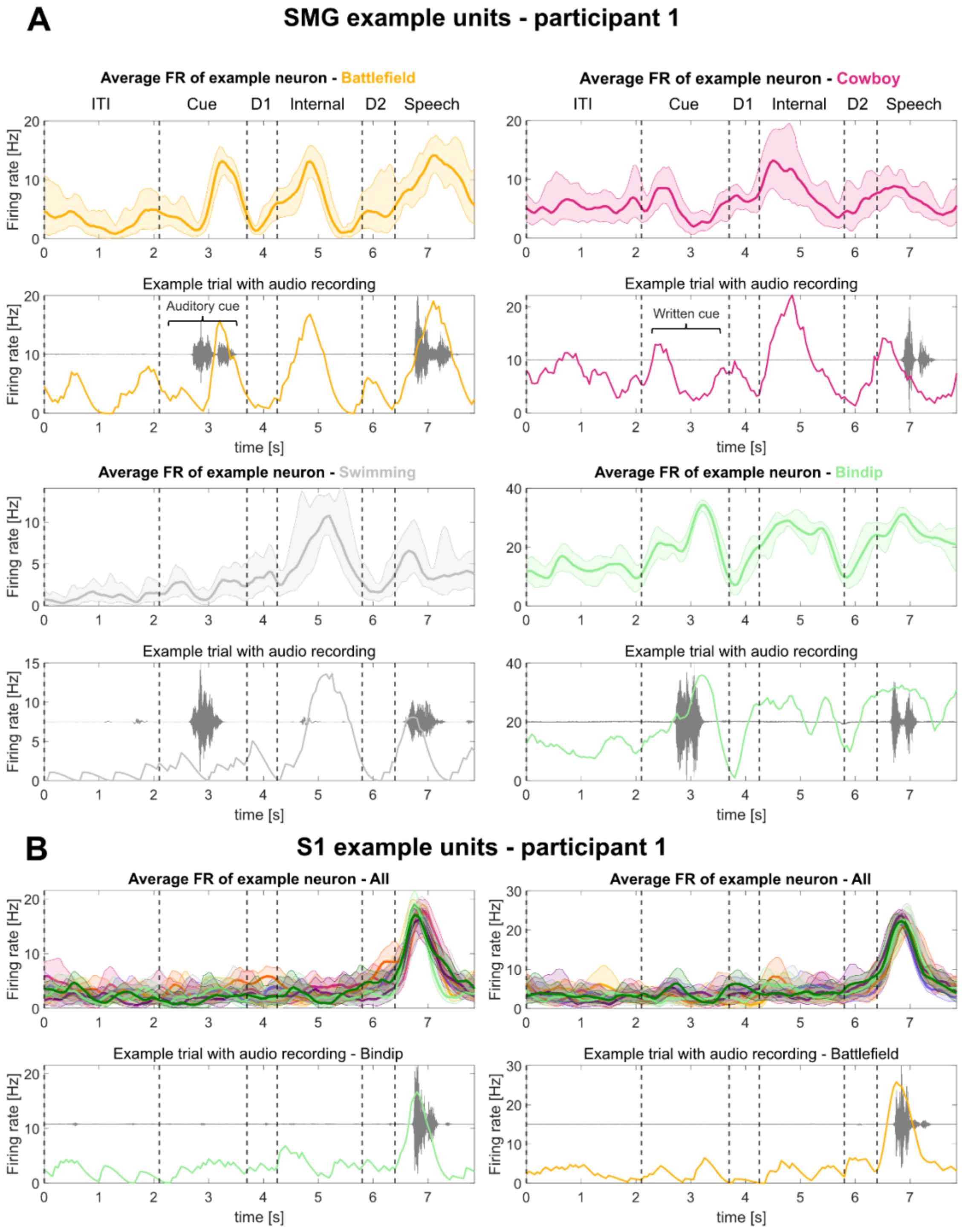

Figure S1 | SMG shows firing rate modulation during cue, internal speech, and vocalized speech, while S1 shows firing rate modulation only during vocalized speech. A) Additional example smoothed firing rates of neurons tuned to four words in SMG for participant 1 during the “Auditory cue” and the “Written cue” task. The top part of each

word figure shows the average firing rate over eight trials (solid line: mean, shaded area: 95% bootstrapped confidence interval). The bottom part of each figure shows an example trial with associated audio amplitude (gray). Vertically dashed lines indicate the beginning of each phase. **B)** Example smoothed firing rates for S1 for participant 1 over task duration. Tuning of a neuron to all words simultaneously was shown to emphasize generalized speech activity to vocalized words (solid line: mean, shaded area: 95% bootstrapped confidence interval for each individual word).

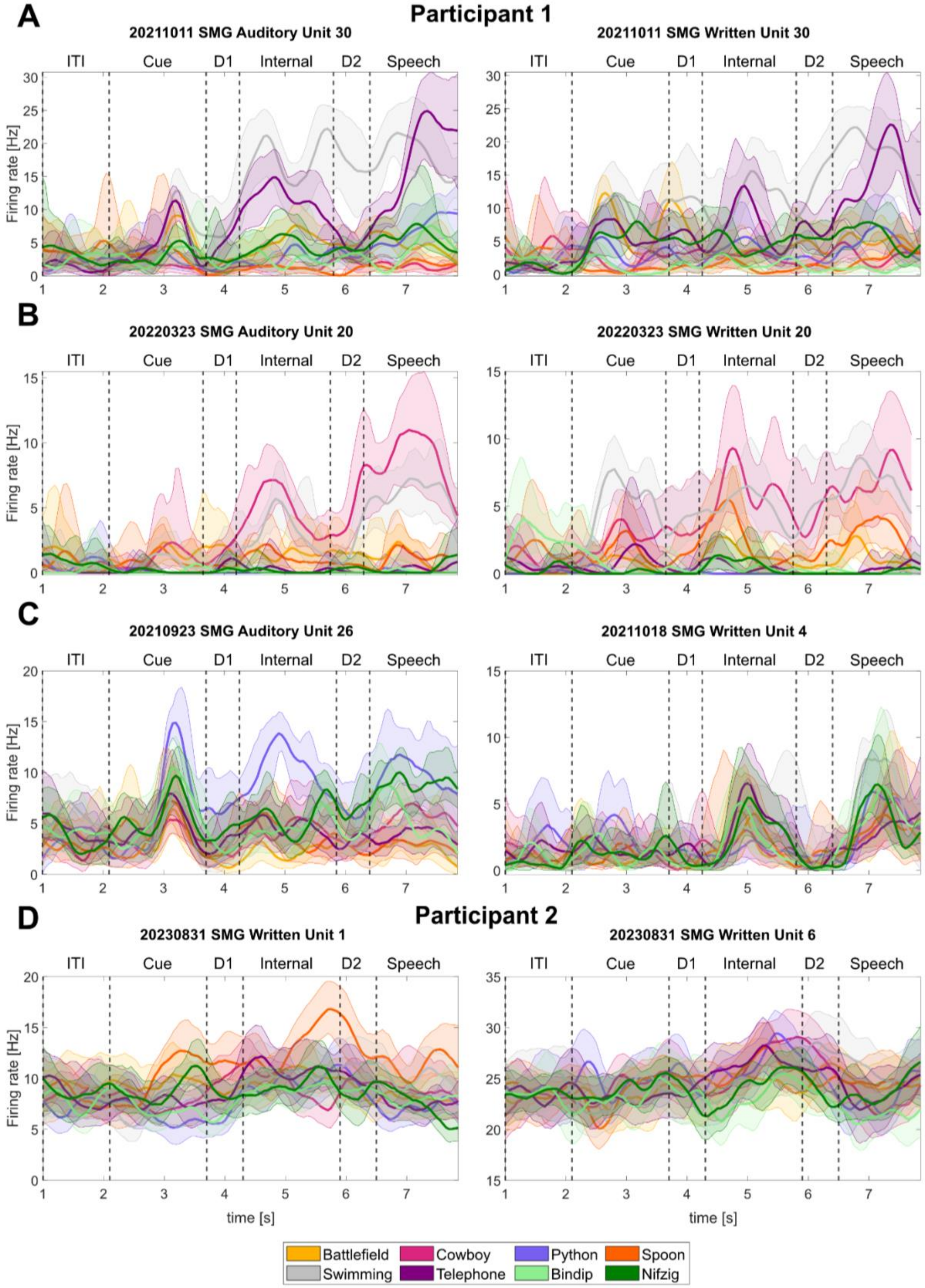
A
Participant 1
20211011 SMG Auditory Unit 30
20211011 SMG Written Unit 30
B
20220323 SMG Auditory Unit 20
20220323 SMG Written Unit 20
C
20210923 SMG Auditory Unit 26
20211018 SMG Written Unit 4
D
Participant 2
20230831 SMG Written Unit 1
20230831 SMG Written Unit 6
ITI
Cue
D1
Internal
D2
Speech
Firing rate [Hz]
time [s]
Battlefield
Cowboy
Python
Spoon
Swimming
Telephone
Bindip
Nifzig

Figure S2 | SMG firing rates for eight words over trial duration. Example smoothed firing rates of neurons tuned to eight words in SMG for participant 1 **(A-C)** and participant 2 **(D)**. Figures shows the average firing rate over eight trials (solid line: mean, shaded area: 95% bootstrapped confidence interval) starting 1 second before cue presentation. A) and B) show tuning of the same neuron on the same day in the “Auditory cue” and the “Written cue” task, demonstrating stable word representation in different task conditions.

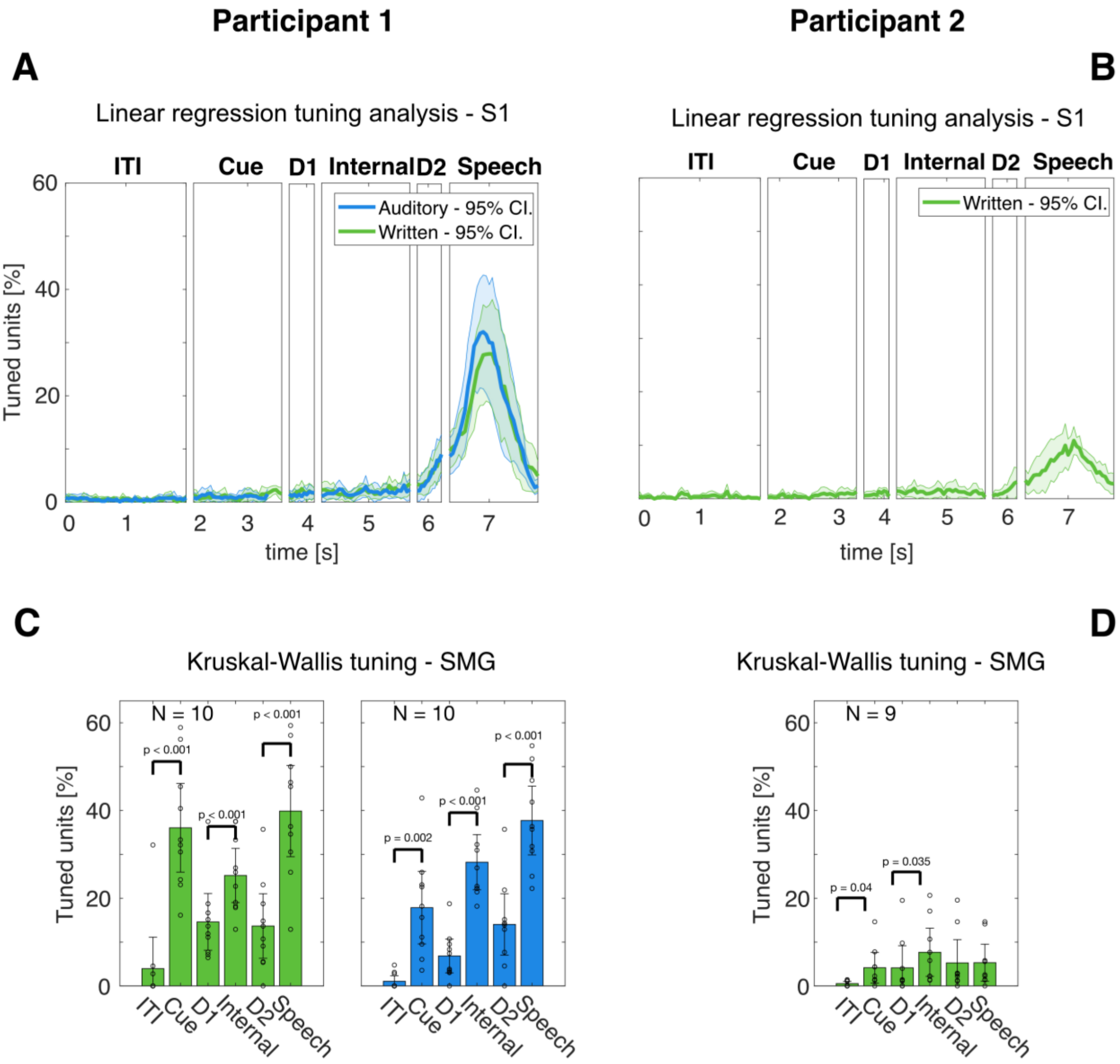

Figure S3 | SMG and S1 tuning analysis. A) Participant 1 S1 tuning analysis through linear regression. Average percentage of tuned neurons to words in 50ms time bins in S1 over the trial duration for "Auditory cue" (blue) and "Written cue" (green) tasks (solid line: mean over 10 sessions, shaded area: 95% confidence interval of the mean). **B)** Same as A) for participant 2, for "Written cue" task with 8 sessions. These results show while lip and face activity are represented in the putative arm area in S1, no activity is elicited during internal speech. **C)** Average percentage of tuned neurons as computed through a Kruskal-Wallis test for each task phase for "Auditory cue" (blue) and "Written cue" (green) tasks (solid line: mean over 10 sessions, 8 trials per condition) for participant 1. Tuning during action phases (Cue, Internal, Speech) following rest phases (ITI, D1, D2) was significantly higher (two-tailed t-test: Written cue: df = 9, $p < 0.001$ for all, Cohen's d >= 1.6 , Auditory Cue: df = 9, $p_{ITI\_Cue} = 0.002$, Cohen's d = 1.36, all others = $p < 0.001$, Cohen's d >= 2.42). **D)** Same as C) for participant 2, with 9 sessions and 16 trials per condition. Tuning during Cue and Internal phases was significantly higher than during rest phases ITI and D1 (two-tailed t-test: df = 8, $p_{ITI\_Cue} = 0.04$, Cohen's d = 0.82, $p_{D1\_Internal} = 0.035$, Cohen's d = 0.84). Tuning during Internal speech phase is significantly different from tuning

during ITI (two-tailed t-test, df = 8, p = 0.015, Cohen's d = 1.02). Word tuning is noticeably lower than in participant 1, leading to subsequently lower classification accuracies.

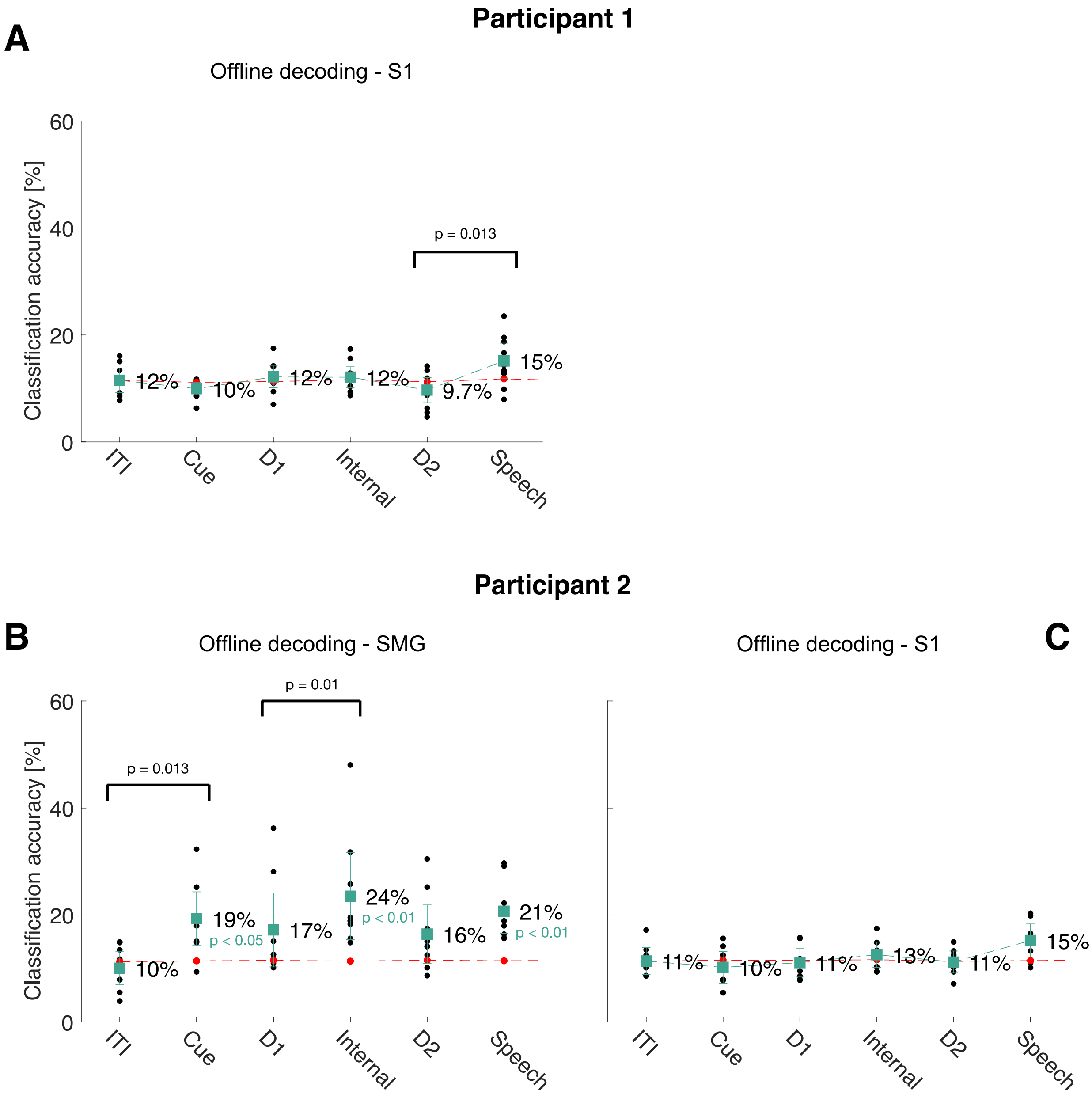

Figure S4 | S1 and SMG offline decoding accuracies. A) Participant 1 S1 decoding analysis. "Auditory cue" and "Written cue" tasks data were combined for each individual session day (~16 trials per word) and leave one out cross-validation was performed (black dots). PCA was performed on the training data, a LDA model was constructed, and results were plotted with 95% c.i, of the session means. Significance of classification accuracies was evaluated by comparing results to a shuffled distribution (averaged shuffle results = red dots). No classification accuracy was significant. However, classification accuracy during vocalized speech was significantly higher than during the previous delay period (paired two-tailed t-test: df = 9, p = 0.013, n = 10). Lack of decoding during the cue phase suggest no auditory contamination occurred in S1 channels**. B)** For participant 2, data of 16 trials per word during the "Written cue" experiment were combined. Confidence intervals and significance were computed as for A. In SMG, significant word decoding was observed during the cue, internal and vocalized speech phases (averaged shuffle results over 100 repetitions = red dots, n = 9, mean decoding value above 97.5 / 99.5 percentile of shuffle distribution = p < 0.01 / p < 0.05, df = 8, per phase Cohen's d = 0.35, 1.15, 1.09, 1.44, 0.99, 1.49, confidence interval of the mean = +/- 3.09, 5.02, 6.91, 8.14, 5.45, 4.15).

Decoding accuracies were significantly higher in the cue and internal speech condition, compared to ITI and D1 (paired two-tailed t-test, n = 9, df = 8, p_{ITI_Cue} = 0.013, Cohen's d = 1.07, $p_{D1\_Internal}$ = 0.01, Cohen's d = 1.11). **C)** S1 decoding mirrored results in participant 1, suggesting no synchronized face movements occurred during the cue phase or internal speech phase. Confidence intervals and significance were performed as for A-B, with n = 8, df = 7).

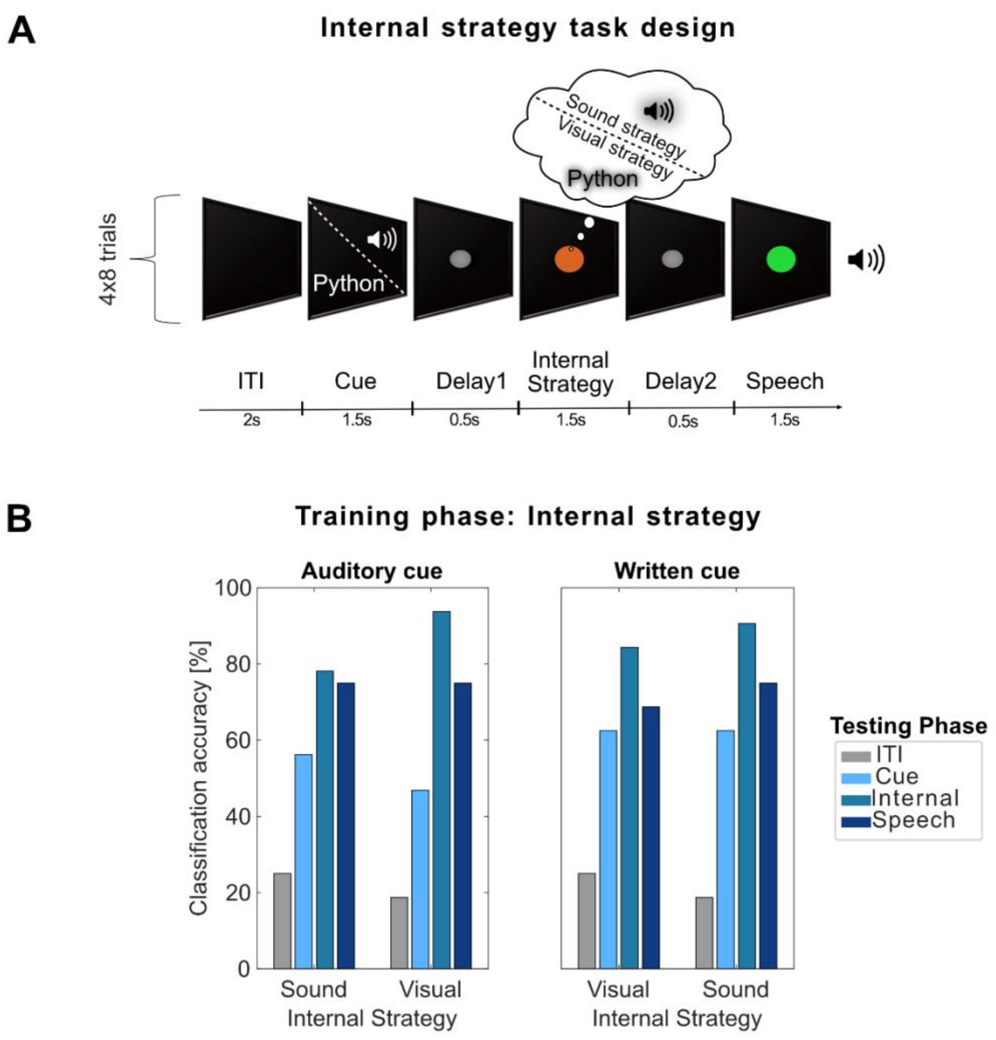

Figure S5 | Different internal speech strategies are represented in SMG. **A)** The task was designed to vary the internal strategy participant 1 was employing during the internal speech phase. Two internal speech strategies were tested: a sound imagination and a visual imagination strategy. For the "sound imagination" strategy, the participant was instructed to imagine the sound of the word. For the "visual imagination" strategy, the participant was instructed to perform mental visualization of the written word. To test if the cue modality (auditory or written) could influence the internal strategy, each internal strategy was run once with an auditory cue, and once with a written cue, resulting in four different task versions (Auditory/Sound, Auditory/Visual, Written/Sound, Written/Visual – see methods). A subset of four words was used for this experiment. **B)** Cross-phase classification was performed by training the model on a subset of data from one phase (e.g. Cue) and applying it on a subset of data from each phase. This analysis was performed separately for each phase, and for each of the four task versions. Plotted here are the results when training on the internal speech phase, and evaluating it on ITI, Cue, Internal, and Speech phases. High classification accuracies (up to 94%) while performing the internal strategy were achieved using both visual and sound imagination strategy.
