# Supplementary material for: Representation of internal speech by single neurons in human supramarginal gyrus
Source: Nat Hum Behav. 2024 May 13;8(6):1136–49. doi: 10.1038/s41562-024-01867-y (PMC11199147; doi:10.1038/s41562-024-01867-y)
Supplement: Supplementary file 2 — Reporting Summary [file 41562_2024_1867_MOESM2_ESM.pdf]

nature portfolio

Corresponding author(s): Sarah Kim Wandelt

Last updated by author(s): Feb 22, 2024

Reporting Summary

Nature Portfolio wishes to improve the reproducibility of the work that we publish. This form provides structure for consistency and transparency in reporting. For further information on Nature Portfolio policies, see our Editorial Policies and the Editorial Policy Checklist.

Statistics

For all statistical analyses, confirm that the following items are present in the figure legend, table legend, main text, or Methods section.

n/a	Confirmed	
☐	☒	The exact sample size (n) for each experimental group/condition, given as a discrete number and unit of measurement
☐	☒	A statement on whether measurements were taken from distinct samples or whether the same sample was measured repeatedly
☐	☒	The statistical test(s) used AND whether they are one- or two-sided<br>*Only common tests should be described solely by name; describe more complex techniques in the Methods section.*
☐	☒	A description of all covariates tested
☐	☒	A description of any assumptions or corrections, such as tests of normality and adjustment for multiple comparisons
☐	☒	A full description of the statistical parameters including central tendency (e.g. means) or other basic estimates (e.g. regression coefficient) AND variation (e.g. standard deviation) or associated estimates of uncertainty (e.g. confidence intervals)
☐	☒	For null hypothesis testing, the test statistic (e.g. F, t, r) with confidence intervals, effect sizes, degrees of freedom and P value noted<br>*Give P values as exact values whenever suitable.*
☒	☐	For Bayesian analysis, information on the choice of priors and Markov chain Monte Carlo settings
☒	☐	For hierarchical and complex designs, identification of the appropriate level for tests and full reporting of outcomes
☒	☐	Estimates of effect sizes (e.g. Cohen's d, Pearson's r), indicating how they were calculated

Our web collection on statistics for biologists contains articles on many of the points above.

Software and code

Policy information about availability of computer code

Data collection	Neuroport System Blackrock Microsystems
Data analysis	Matlab 2022a,b. Python 3.10.8, Psychophysics Toolbox extension for MATLAB (2018)

For manuscripts utilizing custom algorithms or software that are central to the research but not yet described in published literature, software must be made available to editors and reviewers. We strongly encourage code deposition in a community repository (e.g. GitHub). See the Nature Portfolio guidelines for submitting code & software for further information.

Data

Policy information about availability of data

All manuscripts must include a data availability statement. This statement should provide the following information, where applicable:

- Accession codes, unique identifiers, or web links for publicly available datasets
- A description of any restrictions on data availability
- For clinical datasets or third party data, please ensure that the statement adheres to our policy

The custom code developed for this study is openly available at https://doi.org/10.5281/zenodo.10697024. The data supporting the findings of this study are openly available at https://doi.org/10.5281/zenodo.10697024.

Research involving human participants, their data, or biological material

Policy information about studies with human participants or human data. See also policy information about sex, gender (identity/presentation), and sexual orientation and race, ethnicity and racism.

Reporting on sex and gender	These data were not reported for privacy concerns for the participant.
Reporting on race, ethnicity, or other socially relevant groupings	These data were not reported for privacy concerns for the participant.
Population characteristics	These data were not reported for privacy concerns for the participant.
Recruitment	Participants were recruited through our collaborating hospitals according to IRB approved inclusion and exclusion criterion.
Ethics oversight	Institutional Review Board of Rancho Los Amigos National Rehabilitation Center, Institutional Review Board of California Institute of Technology

Note that full information on the approval of the study protocol must also be provided in the manuscript.

Field-specific reporting

Please select the one below that is the best fit for your research. If you are not sure, read the appropriate sections before making your selection.

☐ Life sciences ☒ Behavioural & social sciences ☐ Ecological, evolutionary & environmental sciences

For a reference copy of the document with all sections, see nature.com/documents/nr-reporting-summary-flat.pdf

Behavioural & social sciences study design

All studies must disclose on these points even when the disclosure is negative.

Study description	Data were quantitative experimental. Neuronal firing rates were recorded while a participant performed an internal and vocalized speech task. Recorded data were both analysed offline and in real time.
Research sample	Two human participants affected by tetraplegia performed internal and vocalized speech tasks. For participant 1, an average of 33 sorted SMG units (between 22–56) and 83 sorted S1 units (between 59–96) were recorded per session. For participant 2, an average of 80 sorted SMG units (between 69–92) and 81 sorted S1 units (between 61 – 101) were recorded per session. For participant 1, offline datasets were composed of 8 trials per word across ten sessions. Trials during which participant errors occurred were excluded. In total, between 156-159 trials per word were included, with a total of 1257 trials for offline analysis. Two experimental conditions were run, an Auditory cue condition and a Written cue condition. On four nonconsecutive session days, the Auditory cue task was run first, and on six nonconsecutive days, the Written cue task was run first. For online analysis, datasets were recorded on three different session days, for a total of 304 trials. Participant 2 offline dataset was composed of 16 trials per word using the written cue modality over nine sessions. Error trials were excluded. In total, between 142-144 trials per word were kept, with a total of 1145 trials for offline analysis. For online analysis, datasets were recorded on three session days, leading to a total of 448 online trials.
Sampling strategy	For offline and online experiments, sessions were repeated on different session days. On each session day, results were significantly above chance, demonstrating stable results. Chance was determined by randomizing trial labels 100 times, creating a null distribution. Number of sessions, session length and number of trials was maximized according to participant availability and stamina.
Data collection	Data were recorded with Blackrock Neuroport arrays. Blackrock NSP (Neural signal processor) system and headstages were used. Two researchers were present during all data collection, blindness was not applicable.
Timing	Data were collected between July 2021 and December 2022 for participant 1. For participant 2, data were recorded in January 2023.
Data exclusions	Data were not excluded from the analysis, except if participants were unable to continue experiments due to intense fatigue.
Non-participation	No participant dropped out.
Randomization	Participants were not allocated into experimental groups. In both participant we evaluated internal speech representation in the supramarginal gyrus.

Reporting for specific materials, systems and methods

We require information from authors about some types of materials, experimental systems and methods used in many studies. Here, indicate whether each material, system or method listed is relevant to your study. If you are not sure if a list item applies to your research, read the appropriate section before selecting a response.

Materials & experimental systems

n/a	Involved in the study
☒	☐ Antibodies
☒	☐ Eukaryotic cell lines
☒	☐ Palaeontology and archaeology
☒	☐ Animals and other organisms
☐	☒ Clinical data
☒	☐ Dual use research of concern
☒	☐ Plants

Methods

n/a	Involved in the study
☒	☐ ChIP-seq
☒	☐ Flow cytometry
☒	☐ MRI-based neuroimaging

Clinical data

Policy information about clinical studies

All manuscripts should comply with the ICMJE guidelines for publication of clinical research and a completed CONSORT checklist must be included with all submissions.

Clinical trial registration	NCT01964261
Study protocol	The study protocol is not available to the public, but information is present at clinicaltrials.gov.
Data collection	The first participant was implanted in November 2016. Data were collected between July 2021 and December 2022. Participant 2 was implanted in October 2022. For participant 2, data were recorded in January 2023. Sessions were recorded at participants residence.
Outcomes	We hypothesized that neurons in the supramarginal gyrus modulate to internal speech processed. To evaluate our hypothesis, we recorded from groups of neurons (22–56 per session day for participant 1, 69–92 for participant 2), and evaluated tuning to internally spoken words using a linear regression analysis and a Kruskal Wallis test. Results were compared to a null distribution over 9 and 10 session days respectively. We further hypothesized that we could decode internal speech offline and in real time. Classification performances during internal and vocalized speech processes were evaluated by performing a LDA classifier and to comparing results to a null distribution that involved shuffling labels 100 (offline) or 1000 (online) times on each individual session day.
